# Thyroid Cancer Incidence Rates in North Dakota are Associated with Land and Water Use

**DOI:** 10.3390/ijerph16203805

**Published:** 2019-10-10

**Authors:** Gary G. Schwartz, Marilyn G. Klug

**Affiliations:** Department of Population Health, University of North Dakota School of Medicine & Health Sciences, Grand Forks, ND 58202-9037, USA; marilyn.klug@und.edu

**Keywords:** thyroid cancer, structural equation models, epidemiology, radiation, water

## Abstract

*Objective*: The increasing rate of thyroid cancer diagnoses in the U.S. reflects the increasing use of ultrasonography and of specialist medical care. North Dakota is a rural state with limited access to specialist care, yet its incidence of thyroid cancer is significantly greater than that of the U.S. overall. We sought to identify factors responsible for the high incidence of thyroid cancer in North Dakota. *Methods*: We examined county-specific incidence rates for thyroid cancer in North Dakota in relation to demographic and geographic factors, including median household income, percent of land fertilized, cattle density per capita, and source of drinking water (city or well water), using structural equation modeling. We included county level data on residential radon levels and estimates of radioactive iodine in milk following nuclear weapons testing in the 1950s. *Results*: Thyroid cancer incidence rates were significantly associated with median income (*p* < 0.05); percent of land fertilized (*p* < 0.05); the use of city water (*p* < 0.01), and cattle density per capita (*p* < 0.001). *Conclusions*: The risk of thyroid cancer in North Dakota is positively associated with income and with factors related to land and water use. Our finding that thyroid cancer incidence rates are associated with the use of city water was unexpected and merits examination in other locations with a mix of city and well water use.

## 1. Introduction

Thyroid cancer (TC) represents approximately 1% of all new cancers in men and 4% of new cancers among women. It is the cancer with the fastest increasing incidence rate in the U.S. [1]. During 1975–2009, the incidence of TC in the U.S. nearly tripled; from 4.9 to 14.3 cases per 100,000 per year. Most of the increase in TC diagnoses reflects the increased use of medical imaging, resulting in a higher detection of small, subclinical, papillary tumors that have an excellent prognosis [2]. However, not all of the increase in TC is due to detection bias as there also has been an increase in the incidence of larger, clinically-detected tumors as well as an increase in fatal tumors [3,4].

There are few established risk factors for TC. The best-known factor is exposure to ionizing radiation, especially radiation during childhood [5]. A possible role for ionizing radiation in TC in the U.S. involves nuclear tests conducted by the government during the mid-1950s. Fallout from these tests included radioactive iodine (iodine I-131). The fallout was carried thousands of miles by wind from the Nevada and New Mexico Test Sites and settled on Midwestern pastures. Cattle grazing on these pastures produced radioactive milk and children who consumed that milk incorporated iodine-131 into their thyroids [6]. These children would be expected to have an increased risk of TC as adults, as was reported for a cohort of schoolchildren who lived near the Nevada Test Site in the 1950s [7,8].

**Figure 1:**
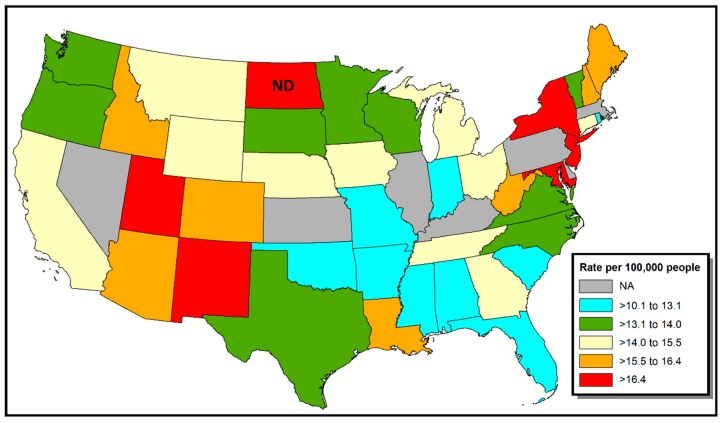
Thyroid cancer incidence in the continental U.S., by state, 2011–2015, for White non-Hispanics. Data are redrawn from State Cancer Profiles [10].

It is likely that thyroid-specific carcinogens other than radiation exist in the environment but have not yet been identified [3]. For example, high TC incidence rates observed in some volcanic regions (e.g., Mt. Etna, Iceland, and Hawaii) led to the hypothesis that heavy metals and/or minerals present in water may cause TC [9]. With regard to geography, a map of cancer incidence in the continental U.S. shows a significantly elevated incidence of TC in North Dakota (ND) (see Figure 1) [10]. For example, the TC incidence rate for White non-Hispanics during 2011–2015 in ND was 17.4 (95% C.I. 15.9–18.9) vs. 15.6 (15.5–15.7) for White non-Hispanics in the U.S. overall.

ND is predominantly rural and has limited access to health care [11]. Thus, the high incidence of TC in ND seems unlikely to reflect detection bias and invites hypotheses as to its cause. We conducted a hypothesis-generating study to gain insight into factors related to TC incidence rates in ND. Because ND is largely agricultural—more than 90% of its area is farmland, we were especially interested in variables associated with agriculture, such as fertilizer use and cattle density [12]. The availability of estimates of the consumption of I-131 milk by county, published by the National Cancer Institute (NCI), provided an opportunity to evaluate a possible role of nuclear fallout.

## 2. Materials and Methods

### 2.1. Data

Data on county-specific incidence rates for thyroid cancer were obtained from the North Dakota Statewide Cancer Registry (NDSCR) for the period 1997–2014. The NDSCR is certified by the American Association of Central Cancer Registries from which it has received awards for data quality and completeness [13]. We used county-specific incidence rates for non-White Hispanics for males and females combined. All rates were age-adjusted to the 2000 Census. In order to prevent disclosure of potentially identifying information, the NDSCR suppresses rates for counties with fewer than 10 cases.

We examined candidate environmental and socioeconomic factors at the county level, including median household income, poverty rate, fertilizer application rates, size of the cattle population, source of drinking water (municipal or self-supplied [i.e., well water]), and sources of radioactivity: uranium levels in soil, indoor radon concentrations, and I-131 levels in milk. The NCI estimated the per capita average radiation does to the thyroid (in rads) per county in the U.S based on the amount of I-131 in milk consumed. Their model is based on wind and weather patterns following the totality of nuclear weapons tests.

Environmental data were extracted from the U.S. Census [14,15]. Data on fertilizer use were obtained from the U.S. Geographic Information Survey [16]. Water use data are from the U.S. Geological Survey [17]. Data on uranium levels in soil are from the U.S. Department of Energy [18]. Radon levels per county are from the state radon monitoring research program conducted in the 1980s, as described previously [19]. Data for I-131 levels for individuals in ND counties were obtained from the NCI website [20]. Because the data for this study came from groups, IRB approval was not required.

### 2.2. Statistical Analysis

We used an approach similar to that of Schwartz et al. in their analysis of colorectal cancer rates [21]. Multivariate linear regression identified relationships between independent variables with TC incidence rates. Structural equation models (SEMs) were then developed using Proc Calis in SAS v 9.4. Briefly, structural equation modeling is an analytic technique that combines multiple regression analysis with factor analysis. SEM models describe the interdependence of a set of variables with the aim of providing a quantitate test of different theoretical models [22]. County specific data were used to estimate covariances between independent variables and paths (prediction estimations) from the independent variables to TC incidence rates.

## 3. Results

During the period 1997 to 2014 (i.e., dating from the inception of the NDSCR), the TC incidence rate for ND overall was 12/100,000 per year (95% C.I. = 11.4–12.6). Incidence rates for approximately half (27) of ND’s 53 counties were suppressed due to < 10 cases/county. Among the 26 counties with reported data, incidence rates varied 3-fold, from a low of 7.5/100,000 (C.I. = 3.7–13.9) in Bottineau County, in north central ND to a high of 22.3/100,000 (C.I. = 10.9–40.8) in Eddy County, in the center of the state.

The SEM depicts covariances between the geographic and demographic variables and between these variables and TC incidence rates. Thus, more populous (i.e., less rural) counties were significantly associated with higher median income and the use of city water and were negatively associated with cattle per capita. The percent of land fertilized was negatively associated with cattle density, reflecting the fact that land devoted to agriculture, located largely in the central and eastern part of the state, differs from the land devoted to ranching, located in the western part of the state. Similarly, levels of I-131 in milk were negatively associated with the percentage of land fertilized and were positively associated with cattle density.

**Figure 2:**
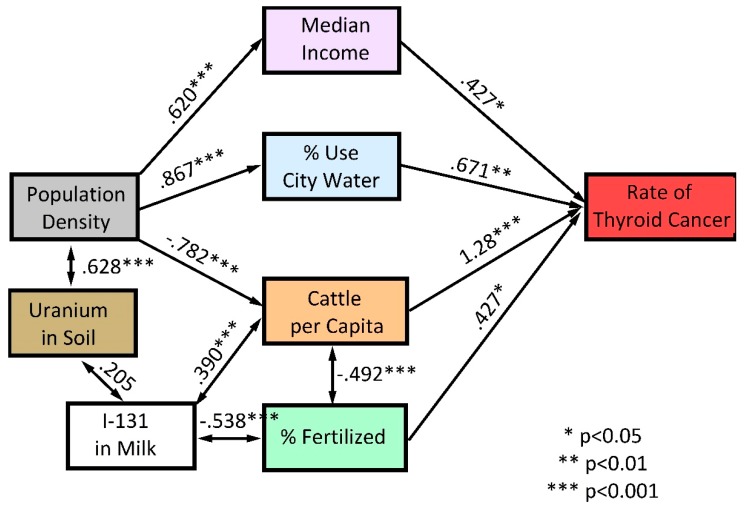
Structural Equation Model for county-specific thyroid cancer incidence rates in North Dakota. Uni-directional arrows indicate potential causal pathways. Bi-directional arrows indicate a co-varying relationship that is unlikely to be causal.

Structural equation modeling identified four variables that were significantly associated with TC rates: median income (*p* < 0.05), percentage of area fertilized (*p* < 0.05), percent of the population using city water (*p* < 0.01), and the density of cattle per capita (*p* < 0.001). Factors such as population density, uranium levels in soil, and I-131 in milk were related to TC indirectly, via their interdependence, and/or through their correlation with income, water source, cattle, and percent acreage fertilized (see Figure 2).

## 4. Discussion

We used SEM as a tool to understand factors that predict county-specific incidence rates of TC within ND. Structural equation modeling captured expected demographic relationships, e.g., that population density is positively associated with median income levels and is negatively associated with cattle density (*p* < 0.001). The model also identified factors with potential causal effects on TC incidence rates: income, cattle per capita, land area exposed to fertilizers, and the use of city water. The model did not identify a significant role for residential radon, a finding in agreement with studies of radon and TC in other U.S. states [23,24].

Our observation of a significant positive effect for income is consistent with a substantial literature indicating that a major contributor to the increasing incidence rates of TC is detection bias [2]. Detection bias would be expected to be more influential in more affluent counties whose residents have greater access to physicians and to screening technologies [25]. In 2015, there were 609 active primary care physicians in ND (population 754,022), yielding ~ 81 primary care physicians per 100,000 residents (vs. 127 per 100,000 for the U.S. overall) [26,27]. Most of these physicians are located in urban areas in ND, areas associated with a higher median income [26].

In the U.S., the detection of TC often begins when a primary care physician observes thyroid nodules in a patient’s neck during a routine physical examination or after X-ray studies performed for other reasons. The nodules typically are evaluated by an endocrinologist via ultrasound and/or biopsy [28]. A recent analysis concluded that half of the interstate variability in incidence rates of TC in the U.S. is explicable by the use of ultrasonography and the number of endocrinologists and general surgeons per capita [29]. In this regard, ND has one of the nation’s lowest numbers of endocrinologists per capita [30]. The low density of primary care and of specialist physicians underscores the view that the high rate of TC in ND is unlikely to result from over-diagnosis.

We found a significant association between TC and counties with a high density of cattle. The “cattle” variable could be a proxy for agricultural exposures that have been reported to be associated with TC, e.g., the use of biocides and/or pesticides [31]. Conversely, it is conceivable that the association of TC with cattle reflects the contamination of pastureland with radioactive I-131 during the Cold War. This interpretation is supported by the significant covariation between cattle density and I-131 doses. A risk from milk consumption would be restricted to individual counties due to the consumption of milk from local cows. This is because the half-life of I-131 is approximately 8 days and the time associated with the processing and shipping of milk would markedly reduce its radioactivity [2,32].

Our analysis identified the percentage of acres fertilized as a significant predictor of TC. Nitrates, which are a major component of fertilizers and of animal manure, have been identified as a risk factor for several cancers, including TC [33,34]. Nitrates in food are converted endogenously to N-nitroso compounds, which are highly carcinogenic [35]. Because nitrates from nitrate-containing fertilizers often contaminate wells, our expectation was that counties with a high proportion of well water use would be positively associated with TC risk [36]. Conversely, we found a significant association with the use of city water. The effect for city water was not due to the association of city water with population density. Population density was included in the SEM model because it is associated with TC risk indirectly (via income, cattle density, and use of city water). Indeed, if population density were allowed in the model as a direct predictor of TC, it would not produce a significant path (*t* = 0.16, *p* = 0.87).

Our finding for city water use raises questions about factors associated with city water that could influence TC risk. City water differs from well water in several respects; the most obvious are that city water is chlorinated and fluoridated. Data on the effects of chlorine on the thyroid are limited. However, studies with human volunteers indicate a lack of significant changes in thyroid hormone levels in adults consuming chlorinated water at a concentrations of 2 and 20 parts per million (mg/L) [37,38]. Conversely, there is evidence that fluoride may cause thyroid dysfunction [39]. Specifically, experimental studies in animals and observational studies in humans have reported alterations in thyroid hormone levels (e.g., T4, T3) and/or an increase in thyroid stimulating hormone (TSH) in individuals consuming fluoridated water [40,41,42]. Increases in TSH thus could promote the growth of latent TCs [43,44]. During the time period 1992–2006, the percentage of North Dakotans receiving city water that was “optimally fluoridated” (defined by the CDC as fluoride levels of 0.7–1.2 ppm) was > 96% (vs. 69.2% for the U.S. overall) [45]. In this regard, significant upregulation of TSH was reported among consumers of fluoridated water with levels as low as 0.5 mg/L [0.5 ppm] [46].

Our study has several limitations. First, this is an ecologic study in which associations were studied at the level of the county. Thus, one cannot conclude that the exposures studied pertain to individuals living in those counties. Secondly, we had no information on possible confounding factors that may modify risk of TC, such as family history or (non-radioactive) iodine intake [47]. Thirdly, the small population size of many ND counties resulted in the suppression of incidence rates for many counties. Excluded counties differ from included counties in several respects, e.g., they have significantly more cattle per capita and a smaller percent of their area fertilized (*p* < 0.05). Conversely, there were no significant differences between included and excluded counties in factors such as the uranium content of soil or in residential radon levels. Although the exclusions may have introduced some bias, the fact that our analyses confirmed established associations with TC, e.g., between TC and income, as well as commonly reported ones, e.g., between TC and exposure to fertilizers, suggests that any bias is unlikely to have distorted the study’s central findings.

Conversely, this study has several strengths. To our knowledge, this is the first report to use data on historic levels of I-131 in milk to explore patterns in TC incidence. The incidence data are population-based, as are the exposure data, which are from the U.S. Census, the U.S. Geological Survey, and other governmental agencies. Data that are of special interest, i.e., on the type of water use (municipal vs. well), likely was determined with high accuracy, as water use is documented by multiple means, including well permits, aerial photography, and real estate and tax records [48]. Our finding that city water use is associated with higher TC risk was unexpected and should be interpreted cautiously. However, it is consistent with several reports implicating fluoride as a cause of thyroid dysfunction and thus merits further investigation.

## 5. Conclusions

TC is the cancer with the most rapidly increasing incidence rate in the U.S. Little is known about its environmental causes. Our structural equation model identified factors relevant to land and water use as significant predictors of TC risk in ND. The positive association observed between TC incidence and the use of city water is a potentially important (albeit provisional) clue that should be examined in other locations that have a mix of city and well water use.
